# Search for small molecule activators of latent HIV

**DOI:** 10.1186/1742-4690-9-S1-O2

**Published:** 2012-05-25

**Authors:** Romas Geleziunas, George Stepan, George Wei, Helen Yu, Michael Graupe, Nikos Pagratis, Tiffany Barnes, Tomas Cihlar, Joe Hesselgesser

**Affiliations:** 1 Clinical Virology at Gilead Sciences, Inc., Foster City, USA

## 

Reservoirs of HIV that persist during ART represent barriers to eradication of this virus. One well documented reservoir of latent HIV is found in memory CD4+ T-cells. Identifying means to safely eliminate latently infected memory CD4+ T-cells is an important goal that may contribute to a cure for HIV. One approach toward this end is to activate latent proviruses with the premise that viral particles emanating from these cells will cause a cytopathic effect leading to the demise of the host cell. We have optimized and automated a primary cell-based HIV latency assay that can be used for high throughput screening of small molecule libraries in search of HIV activators. Using this assay, we have identified novel histone deacetylase (HDAC) inhibitors fromGilead’s compound collection that activate latent HIV. Analysis of these inhibitors revealed that the magnitude of HIV expression correlated with the breadth of cellular HDAC inhibition. In addition, we have identified a variety of other compounds that activate latent HIV such as kinase inhibitors which may point to novel mechanisms that govern HIV latency. This screening assay has the potential to identify novel molecular targets for drug discovery and new chemical classes that could be optimized to create new drugs to eliminate reservoirs of latent HIV.

